# Personal Health Data Tracking by Blind and Low-Vision People: Survey Study

**DOI:** 10.2196/43917

**Published:** 2023-05-04

**Authors:** Jarrett G W Lee, Kyungyeon Lee, Bongshin Lee, Soyoung Choi, JooYoung Seo, Eun Kyoung Choe

**Affiliations:** 1 College of Information Studies University of Maryland College Park, MD United States; 2 Department of Computer Science University of Maryland College Park, MD United States; 3 Microsoft Research Redmond, WA United States; 4 Department of Kinesiology and Community Health University of Illinois at Urbana-Champaign Urbana, IL United States; 5 School of Information Sciences University of Illinois at Urbana-Champaign Champaign, IL United States

**Keywords:** personal health data, self-tracking, blind and low vision, survey, consumer health information, mobile phone

## Abstract

**Background:**

Personal health technologies, including wearable tracking devices and mobile apps, have great potential to equip the general population with the ability to monitor and manage their health. However, being designed for sighted people, much of their functionality is largely inaccessible to the blind and low-vision (BLV) population, threatening the equitable access to personal health data (PHD) and health care services.

**Objective:**

This study aims to understand why and how BLV people collect and use their PHD and the obstacles they face in doing so. Such knowledge can inform accessibility researchers and technology companies of the unique self-tracking needs and accessibility challenges that BLV people experience.

**Methods:**

We conducted a web-based and phone survey with 156 BLV people. We reported on quantitative and qualitative findings regarding their PHD tracking practices, needs, accessibility barriers, and work-arounds.

**Results:**

BLV respondents had strong desires and needs to track PHD, and many of them were already tracking their data despite many hurdles. Popular tracking items (ie, exercise, weight, sleep, and food) and the reasons for tracking were similar to those of sighted people. BLV people, however, face many accessibility challenges throughout all phases of self-tracking, from identifying tracking tools to reviewing data. The main barriers our respondents experienced included suboptimal tracking experiences and insufficient benefits against the extended burden for BLV people.

**Conclusions:**

We reported the findings that contribute to an in-depth understanding of BLV people’s motivations for PHD tracking, tracking practices, challenges, and work-arounds. Our findings suggest that various accessibility challenges hinder BLV individuals from effectively gaining the benefits of self-tracking technologies. On the basis of the findings, we discussed design opportunities and research areas to focus on making PHD tracking technologies accessible for all, including BLV people.

## Introduction

Over the last decade, technologies for individuals to track personal health data (PHD; eg, exercise, sleep, diet, blood pressure, and blood glucose) have become increasingly popular. The number of mobile health (mHealth) and fitness apps has increased, reaching >350,000 in 2021 [1]. Approximately 1 in 5 Americans are currently using fitness trackers including smartwatches, and a similar number of Americans are using health and fitness apps on mobile devices [2]. People with or without health problems use these technologies to manage their health and well-being. Many studies have shown the benefits of personal health technologies in monitoring health status, managing chronic diseases, and maintaining healthy lifestyles [3-7].

However, these technologies are often designed for sighted people using graphical user interfaces (GUIs) and providing vision-dependent feedback to aid in data collection and reflection. For example, commercial food and nutrition tracking apps (eg, MyFitnessPal and Love It!) use vision-oriented designs to capture data (eg, photo journaling and barcode scanning) that are not designed with blind and low-vision (BLV) people in mind. These apps often provide feedback through data visualizations (eg, bar, line, and pie charts) without providing adequate text descriptions. In addition, many extra features and advertisements of the app put an additional burden on blind people who cannot easily navigate or ignore them.

As such, personal health tracking technologies remain largely inaccessible to the BLV population, providing limited self-tracking experience [8]. Therefore, it is imperative for accessibility researchers and designers to understand the first-hand self-tracking experiences and identify unique self-tracking needs and accessibility challenges of BLV people. Recent studies have started looking into accessibility barriers of mHealth apps, but they either casted a wide net covering all types of disabilities instead of focusing on BLV [9-11] or examined accessibility issues of only a few selected mHealth apps [8,12,13], limiting the generalizability of the findings.

This study aims to fill this gap by gaining an in-depth understanding of why and how BLV people collect and use their PHD and the obstacles they face. We reported the results from a web-based and phone survey we conducted to understand BLV people’s current practices and lived experiences of tracking and using their PHD. We also discussed the implications for designing and developing personal health technologies that are accessible to BLV people. Throughout this paper, we use the identity-first language (ie, BLV people) instead of person-first language (ie, people who are blind or have low vision [LV]) because both our community partner, National Federation of the Blind, and the blind author of this paper view blindness as identity rather than any negative characteristic that defines one or one’s future.

Health disparities emerge from health system disparities and socioeconomic factors that create differential access to resources [14]. It is now generally accepted that health and health care are strongly influenced by race, ethnicity, socioeconomic status, age, sex, residence, and social networks [15,16]. Although the relationship between digital technologies and health is nonlinear and complex, digital health ecosystems can deepen the existing health disparities between people with and without disabilities [17].

Although many digital technologies, including wearable tracking devices and mobile apps, have great potential to equip the general population with the ability to monitor and manage their health [18], they could also threaten the equitable access to PHD and health care services among people with disabilities [19]. In other words, the inaccessible designs and poor usability of digital technologies that were originally designed to support individuals’ health care may widen the existing health disparity gap in our society [10,20].

People with disabilities often seem to be marginalized from the benefits of digital health technologies. Jones et al [10] conducted a survey with people with all types of disabilities about their experiences of using personal health apps. They found that people with disabilities have strong needs and desires to use mHealth apps (ie, 40% of current users) but face many challenges including difficulty locating suitable apps and accessibility problems.

In studies specifically targeting BLV people, researchers investigated the accessibility of personal health apps [8,12,21]. Although sighted people use personal health technologies to stay healthy, the available options and benefits from those technologies are limited for BLV people [21]. It is especially challenging for BLV people to learn how to use highly visually oriented technologies compared with those with other types of disabilities [22]. Milne et al [8] assessed the accessibility of 9 mHealth apps (5 blood glucometer apps and 4 blood pressure apps) and found that none of them met the basic accessibility guidelines. Kim [12] checked the level of accessibility of the 5 most commonly used self-care apps using the Web Content Accessibility Guidelines 2.0 mobile accessibility checklists [12]. For each of the Web Content Accessibility Guidelines accessibility principles, they identified several issues, including information overload, no access to GUIs, and incomplete (or missing) description about user interface components.

Although previous works point to high-level accessibility pitfalls of mHealth technologies, we still lack an understanding of the in-depth accessibility challenges BLV people have faced in adopting and using mHealth technologies. To fill this gap, we investigated BLV people’s experience of tracking and using their PHD through a comprehensive web-based and phone survey.

## Methods

### Overview

We conducted a web-based survey on Qualtrics [23] to understand BLV people’s current practices and lived experiences of tracking and using their PHD. We ensured that our survey was accessible to screen readers (eg, JAWS, NVDA, and VoiceOver), the main assistive technology tools for BLV people that narrate digital content as synthesized speech.

### Survey Design

We started the survey with questions about basic background information, such as the vision history and status, list of devices they owned, self-reported health conditions, and the level of interest in their own health. Before asking questions about PHD, we defined the term “Personal Health Data” as health-related data that people can track about themselves outside the clinic [7] and provided examples, such as exercise (eg, step count and miles run), sleep, diet, heart rate, weight, and blood pressure data. As we wanted to ask different sets of questions based on people’s experience with PHD tracking, we asked a question to categorize respondents into 1 of the 3 groups: current tracker (CT), past tracker (PT), and no experience (NE).

Our main survey asked about four aspects of PHD tracking experience: (1) types of data, (2) tracking methods and tools, (3) reasons for tracking (or not), and (4) barriers to tracking. For the data types and tracking methods and tools, we asked respondents to select items from a comprehensive list and let them also specify any missing items using the “Others” option. We used open-ended questions to elicit the reasons for tracking (or not) and barriers to tracking. Textbox 1 presents the key questions for each aspect; we have slightly rephrased some of the questions for different groups based on the respondents’ answers to prior questions. A full list of questions can be found in Multimedia Appendix 1.

**Key questions asked in our survey.**

**Types of personal health data**

- What types of personal health data do you currently (or did you formerly) track? (Check all that apply)
- In addition to what you have been tracking, what other personal health data do you want to track, if any? (Check all that apply)

**Tracking methods and tools**

*For current tracker (and past tracker)*

- How do (did) you track your personal health data, if any? (Check all that apply)
- *If smartphone apps or wearable devices are selected*
  What smartphone apps (or wearable devices) have you used to track which of your data?
  How was your experience?
- How did you access the data you tracked? (eg, screen reader, smartphone’s text to speech, or ask for help)? Did you have any accessibility challenges when accessing your data?

*For no experience*

- If you wanted to track your health data, what would your preferred method be? (Check all that apply)

**Reasons for tracking (or not)**

- Why are you tracking your personal health data?
- Why did you stop tracking your personal health data?
- Could you explain why you do not want to collect your personal health data?

**Barriers to tracking**

- Please describe any barriers or challenges you faced when tracking your personal health data.

### Respondents’ Recruitment

We recruited people who were interested in PHD and met the following inclusion criteria: people who were (1) blind or LV (ie, those with mild to severe distance vision impairment); (2) aged at least 18 years; and (3) able to participate in the survey via a computer, mobile device, or phone. We provided an option to take the survey over the phone, in which case a researcher read out the questions and recorded responses. We recruited respondents via the National Federation of the Blind’s mailing list, researchers’ personal network, and web-based BLV communities on Facebook. The survey was available on the web during April and May 2022. As it was created in English, the survey was accessible to those who spoke English.

### Data Analysis

We initially had a total of 203 (198 via the web and 5 via phone) survey responses. Of the 184 completed responses, we excluded 2 (1.1%) that were submitted from the same IP address. On further inspection of the quality of the responses, we noticed a suspicious data entry pattern. A total of 26 responses repeated the same answer (“No,” “n,” or “I don’t really want to say”) for several open-ended optional questions, with most having the same set of answers across several response IDs. As it is highly unlikely that different individuals submit the same answers for multiple open-ended questions that are optional, we excluded these from our data set, resulting in a total of 156 responses.

We used open coding to analyze qualitative data from open-ended survey responses. Through several rounds of iterative refinements, we generated 71 codes that captured representative ideas in the data. We then used affinity diagramming to categorize similar codes and drew higher-level themes over several refinements. For quantitative data, such as demographics, PHD type, and data capturing methods, we characterized the data using descriptive statistics.

### Ethics Approval

Our survey study was reviewed and approved by the University of Maryland, College Park’s Institutional Review Board under #1854542. We maintained the confidentiality of our respondents by using a security-accredited survey provider, deidentifying survey results during our analysis phase and in our reporting, and solely using password-protected servers and workstations for data storage.

To motivate participation, we used raffles, offering a US $50 Amazon gift card to 2 respondents and a US $20 Amazon gift card to 10 respondents. Before individuals took our survey, they were informed of our study’s procedures, potential risks and benefits, and their individual rights as participants and were given the opportunity to opt-out.

## Results

### Respondents’ Demographics and Tracking Background

Table 1 summarizes the demographic characteristics of 156 respondents. Nearly an equal number of respondents who self-identified as totally blind (TB; 79/156, 50.6%) or LV (77/156, 49.4%) participated in our survey. Approximately two-thirds of them were CT (102/156, 65.4%), 19.2% (30/156) were PT, and 15.4% (24/156) had NE tracking PHD. More than half of the respondents were visually impaired since birth (83/156, 53.2%), and approximately one-third became visually impaired after birth and had been visually impaired for at least 10 years (51/156, 32.7%). Most respondents (151/156, 96.8%) rated their health condition as fair or better. Respondents varied in age, with 22.4% (35/156) aged 35 to 44 years and 21.2% (33/156) aged 55 to 64 years; 68.6% (107/156) were female, 29.5% (46/156) were male, and 1.9% (3/156) were nonbinary. More than two-thirds of the respondents were White (107/156, 68.6%), 10.9% (17/156) were Asian, and 5.8% (9/156) were Black or African American. In terms of education, 59.6% (93/156) of the respondents had a bachelor’s degree or higher.

**Table 1:** The demographic and technology ownership information of the 156 respondents.

		Totally blind (n=79), n (%)	Low vision (n=77), n (%)	Combined (N=156), n (%)
**Tracking status**				
	Current tracker	54 (35)	48 (31)	102 (65.4)
	Past tracker	15 (10)	15 (10)	30 (19.2)
	No experience	10 (6)	14 (9)	24 (15.4)
**Vision history**				
	Since birth	45 (29)	38 (24)	83 (53.2)
	10 years or longer (but not since birth)	27 (17)	24 (15)	51 (32.7)
	5 years or longer and less than 10 years	4 (3)	8 (5)	12 (7.7)
	1 year or longer and less than 5 years	2 (1)	6 (4)	8 (5.1)
	Less than 1 year	1 (1)	0 (0)	1 (0.6)
	Prefer not to say	0 (0)	1 (1)	1 (0.6)
**Health status**				
	Very poor	1 (1)	1 (1)	2 (1.3)
	Poor	1 (1)	2 (1)	3 (1.9)
	Fair	23 (15)	23 (15)	46 (29.5)
	Good	35 (22)	33 (21)	68 (43.6)
	Very good	19 (12)	18 (12)	37 (23.7)
**Age (years)**				
	18-24	5 (3)	5 (3)	10 (6.4)
	25-34	13 (8)	14 (9)	27 (17.3)
	35-44	19 (12)	16 (10)	35 (22.4)
	45-54	12 (8)	15 (10)	27 (17.3)
	55-64	14 (9)	19 (12)	33 (21.2)
	≥65	15 (10)	7 (4)	22 (14.1)
	Prefer not to say	1 (1)	1 (1)	2 (1.3)
**Gender**				
	Male	16 (10)	30 (19)	46 (29.5)
	Female	62 (40)	45 (29)	107 (68.6)
	Nonbinary or third gender	1 (1)	2 (1)	3 (1.9)
**Ethnicity**				
	White	52 (33)	55 (35)	107 (68.6)
	Asian	12 (8)	5 (3)	17 (10.9)
	Black or African American	5 (3)	4 (3)	9 (5.8)
	American Indian or Alaska Native	3 (2)	2 (1)	5 (3.2)
	Native Hawaiian or other Pacific Islander	0 (0)	1 (1)	1 (0.6)
	Other	6 (4)	9 (6)	15 (9.6)
	Prefer not to say	1 (1)	1 (1)	2 (1.3)
**Education**				
	Less than a high school diploma	1 (1)	0 (0)	1 (0.6)
	High school degree or equivalent	7 (4)	8 (5)	15 (9.6)
	Some college, no degree	18 (12)	15 (10)	33 (21.2)
	Associate degree	6 (4)	8 (5)	14 (9)
	Bachelor’s degree	19 (12)	17 (11)	36 (23.1)
	Master’s degree	25 (16)	22 (14)	47 (30.1)
	Professional degree	1 (1)	2 (1)	3 (1.9)
	Doctorate	2 (1)	5 (3)	7 (4.5)
**Technology owned**				
	Laptop or desktop	78 (50)	73 (47)	151 (96.8)
	Smartphone	73 (47)	74 (47)	147 (94.2)
	Smartwatch or fitness band	37 (24)	32 (21)	69 (44.2)
	Bodyfat or weight scale	36 (23)	33 (21)	69 (44.2)
	Tablet	24 (15)	42 (27)	66 (42.3)
	Medical device	32 (21)	31 (20)	63 (40.4)
	Other	7 (4)	8 (5)	15 (9.6)

### Tracking Items and Data Collection Tools

**Figure 1:**
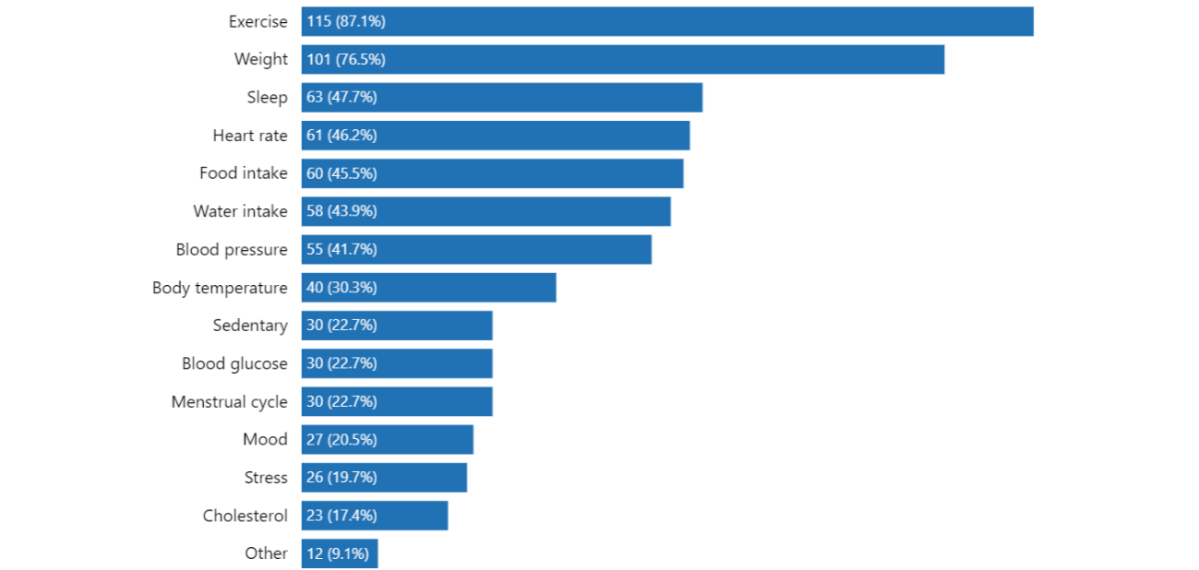
What personal health data current and past tracker respondents (n=132) have been tracking or have tracked.

**Figure 2:**
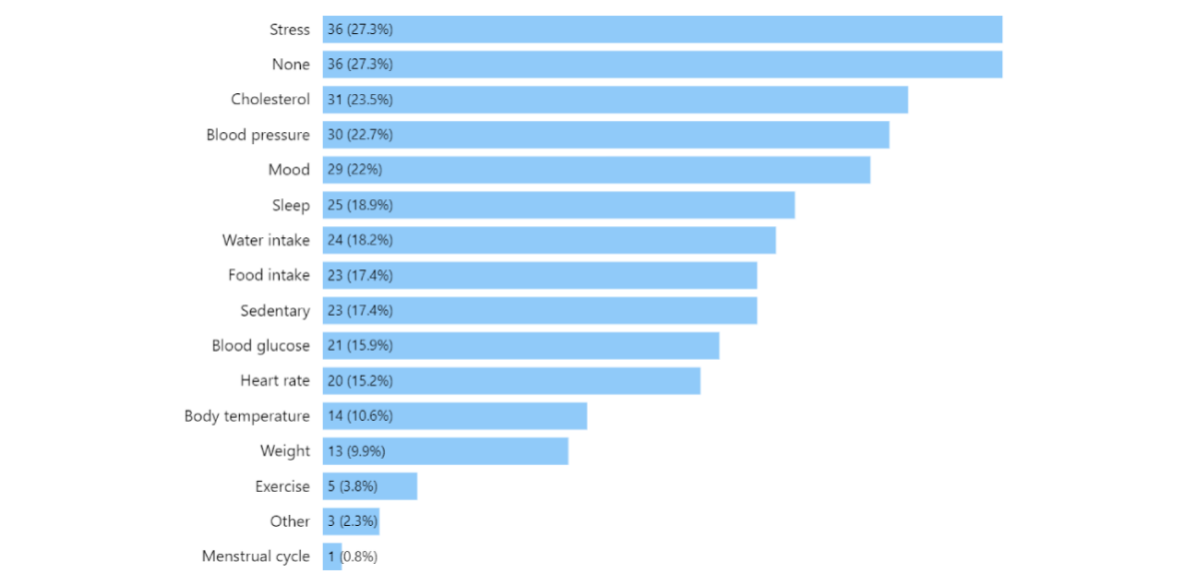
What personal health data current and past tracker respondents (n=132) want to track that they are not currently tracking or have not tracked.

Exercise (115/132, 87.1%) and weight (101/132, 76.5%) were the 2 most popular items the CT and PT respondents reported tracking (Figure 1). Other highly ranked items were sleep (63/132, 47.7%), heart rate (61/132, 46.2%), food (60/132, 45.5%), water (58/132, 43.9%), and blood pressure (55/132, 41.7%). On average, CT respondents tracked 5.8 (SD 2.9; range 1-14) items, and PT respondents tracked 4.7 (SD 2.6; range 1-11) items. In responding to the question “What other PHD do you want to track?,” the CT and PT respondents commonly mentioned stress (36/132, 27.3%), cholesterol (31/132, 23.5%), blood pressure (30/132, 22.7%), and mood (29/132, 22%). We noted that 27.3% (36/132) of respondents selected “None (I don’t have anything else I want to track),” which is tied with “stress” for the most selected. Refer to Figure 2 for further details.

**Figure 3:**
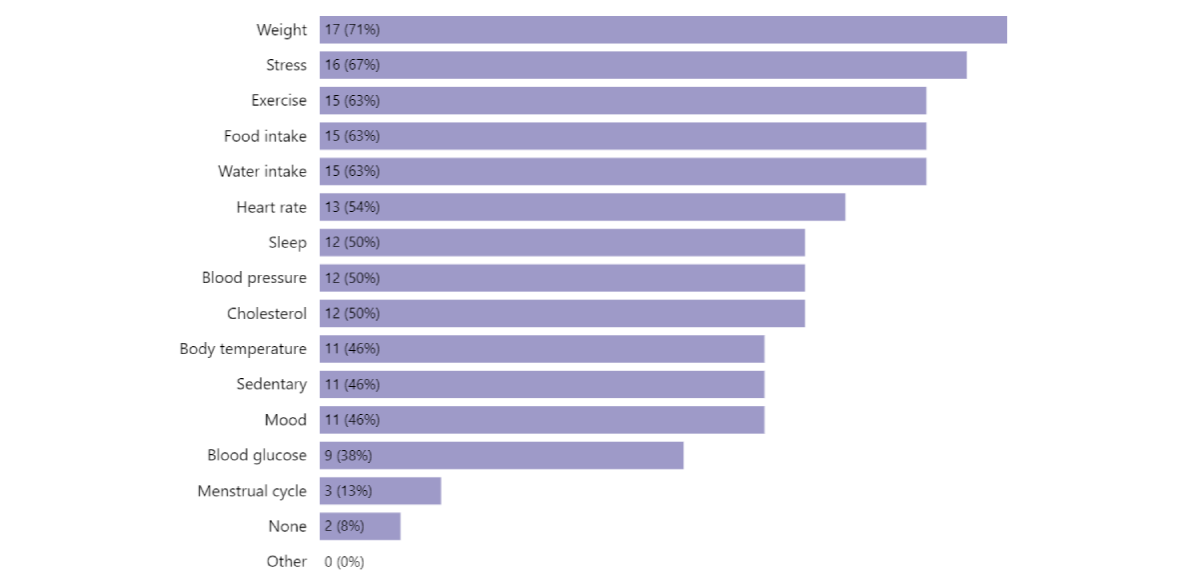
What personal health data no experience (no tracking experience) respondents (n=24) want to track.

For 24 NE respondents, we asked what type of PHD they wanted to track. The top 5 items were weight (17/24, 71%), stress (16/24, 67%), exercise (15/24, 63%), food intake (15/24, 63%), and water intake (15/24, 63%; Figure 3). NE respondents wanted to track 7.3 (SD 4.2) items on average, demonstrating high interest in tracking PHD.

**Figure 4:**
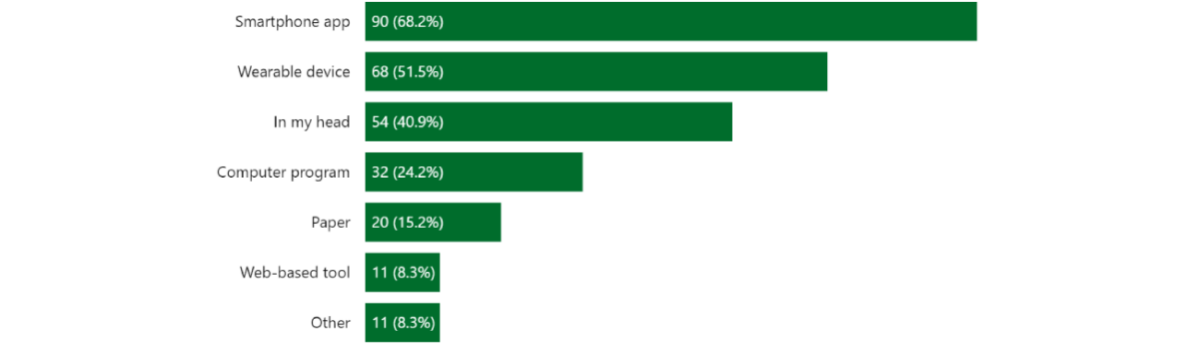
Tracking methods that current and past tracker respondents (n=132) used.

**Figure 5:**
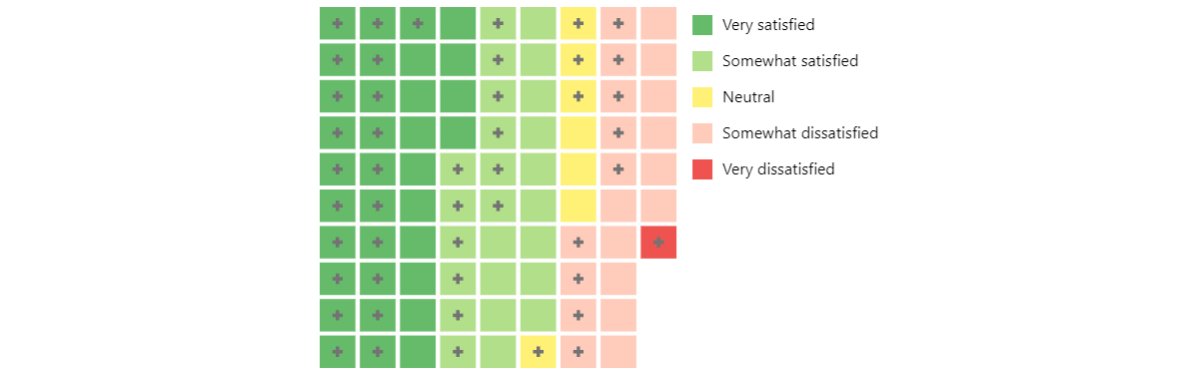
A waffle chart of current tracker respondents (n=87) reporting their satisfaction level toward personal health tracking technology. A cross symbol (+) indicates a totally blind respondent, and color represents a satisfaction level: very satisfied (n=34), somewhat satisfied (n=25), neutral (n=7), somewhat dissatisfied (n=20), and very dissatisfied (n=1).

Figure 4 shows the tracking methods for the CT and PT respondents. A majority of participants used smartphone apps (90/132, 68.2%), with the most frequently mentioned apps being Apple Health (n=45), Fitbit (n=15), and MyFitnessPal (n=15). However, few respondents also mentioned Weight Watchers (n=4); Google Fit (n=3); and Strava (n=3), an outdoor cycling app. Approximately half of the CT and PT respondents reported using wearable devices (68/132, 51.5%); Apple Watch was the most popular device (n=41), followed by Fitbit (n=22). Among the CT who used some sort of technology for tracking (n=87), 59 (68%; 34 very satisfied and 25 somewhat satisfied) respondents reported that they were satisfied with the current technology (Figure 5).

### BLV’s Desire and Needs for Tracking

Our survey with BLV individuals shows that there is not only substantial interest but also important needs for tracking PHD by the respondents (Textbox 2). Unless otherwise noted, the total sample size for this section was 156 respondents.

A total of 33 respondents perceived tracking as an important tool for preventative health. Of this group, 23 described tracking as a means of maintaining their current state, whether sustaining a healthy status or preventing deterioration into a health problem. Awareness of the medical issues in their family’s history and aging were also listed as reasons why tracking was needed.

A means to improve oneself was another desired benefit for trackers. For some, improving one’s health was noted as a step to external goals, such as enabling new activities like aerial arts. General health improvement was a tracking reason for 24 respondents, with weight loss being the most mentioned. Some shared additional benefits of tracking, which helped them toward better health, such as being coached with “*information to figure out how [to] effectively manage my health*.” Tracking systems also provided welcomed encouragement that helped respondents remain on their health journey. For example, 1 respondent whose data led to a sustained effort said the following (as a respondent’s anonymized ID, we used the last 4 letters from the identifier the survey system automatically creates. We used “CT” to denote participants who are “current trackers” and “TB” to denote those who are “totally blind”):

I find that by keeping track of steps, hours standing, heart rate [and] these other issues I can encourage myself to remain active. I can encourage myself to keep on that eating plan or that exercise plan that will help me get to a healthier lifestyle. Seeing the small changes and seeing the trends of how I am improving helps to motivate me to keep going and to start pushing myself to do better.

i6fh, CT and TB

A total of 50 respondents wished to use tracking as a means to learn about themselves, whether simply because of curiosity, or to view what progress was made in their activity. Of this group, the majority (34/50, 68%) found tracking to be an important diagnostic tool for their health, keeping track of various measurements and being made aware of any outlying incidents. Several respondents also valued the averages provided in the tracking systems for measurement comparison.

A group of 22 respondents expanded their analysis and wished to use tracking data for outcome analysis to examine how different actions affected their health. This ranged from inspecting the correlations of activity toward health to the effectiveness of their medications and treatments. One respondent shared how tracking was used to validate the proper use of medical equipment:

With sleep apnea, I need to make sure my mask seal is correct and my number of episodes per hour are below a certain level to gauge whether the treatment is working as it should.

pu8s, CT and TB

**A summary of blind and low-vision respondents’ motivation and needs to track personal health data (PHD).**

**Tool for preventative health**

- To sustain healthy status
- To prevent health deterioration
- To be aware of family- or aging-related issues

**A means to improve oneself**

- Enabling general health improvement, especially weight loss
- Activity as a step toward external goals, for example, losing weight so that one can try new activities
- Learning from system-provided health coaching
- Being inspired by encouragement given by system

**Way to learn about themselves**

- Diagnostic tool for health, for example, blood sugar monitoring
- Allowing a comparison of PHD with system’s global averages
- Tracking personal health activity progress
- Mere curiosity

**Outcome analysis**

- Inspecting correlations of actions toward health
- Measuring the effectiveness of medications and treatments
- Using systems as a historical record keeper

**With and for others**

- Enabling social activities, for example, step count challenges
- Remaining healthy to continue caring for others
- Maintaining health to lessen the need for others’ aid

A total of 13 respondents mentioned the additional use of tracking systems as a historical record keeper as a reason for tracking. Of the 13 respondents, 2 saw this historical data as a means of sharing information with their doctors.

Different aspects of using tracking data with others were mentioned, such as social activities, including step count challenges. A total of 3 respondents tracked PHD to benefit others, striving to remain healthy to continue caring for family or clients in their lives, or merely to lessen the need for others’ aid.

### Barriers to Tracking

#### Overview

In this section, we report a wide range of barriers experienced by BLV respondents in tracking PHD (Textbox 3). Unless otherwise noted, the total sample size for this section is 132 CT and PT respondents.

**Summary of the barriers experienced by blind and low-vision (BLV) respondents in tracking personal health data.**

**Knowing it could be better**

- Blocked from initiating tracking
  No accessible options for tracking certain activities
  Visual roadblocks prevent users from initiating tracking, for example, inaccessible device pairing codes
  Lacking information on accessibility of available apps and devices
  Accessible equipment can be unaffordable for the population
- Adaptation tools not supported by systems
  Incompatibility with tools, for example, screen reader and braille output
  Lack of visibility options, for example, magnification or contrast
  Misapplied aid, for example, screen reader compatible but giving incorrect label for button
- Missing out on features because of disability
  Limited from features on devices because of lack of accessibility, for example, screens on wearables
  Inaccessibility when graphs are used to portray data in apps
  Specific app features not enabled with accessibility
- Information delay
  Waiting for data to be transferred to accessible app
  Waiting for aid of others in reading data
- Negative awareness of sighted users’ experiences
  Measuring time expenditure against sighted users’ efforts
  Jealousy in how others benefit from features inaccessible to them
  Comparison of prior experience to current use with declined vision
  Recognizing accessibility deterioration, for example, losing accessibility functions with new updates

**Perceived low valuation in tracking**

- Tracking can be burdensome for BLV users
  Increased time consumption in entering data and using app
  Needing to involve others to complete tracking procedure
  Data comprehension requiring in-memory manipulation
- Data are not seen as trustworthy
  Observing discrepancies between devices
  Noticing incomplete data about known activities
  Incompatibility of trackers with ailments, for example, cerebral palsy affecting step count
  Not finding discrete data points as representative of true health
- Data elicits negative emotions
  Data becoming too overwhelming
  Privacy concerns
  Lucidity of recorded behavior is upsetting, for example, realization in food tracking
- General disinterest in tracking
  Tracking follows activity—fading when user’s interest in activity fades
  Tracking only seen as an assignment, ending when directive is completed
  Data not providing insight or usefulness

#### Knowing It Could Be Better

Respondents not only noted their suboptimal tracking experiences but also the specific ways in which the situations could be better. This led to frustration and occasional discontinuance of tracking.

Some attempts in tracking were simply blocked at the start. A total of 15 respondents did not have accessible options in trackers, especially for blood glucose and blood pressure monitoring. Tracking procedures requiring vision, such as reading caloric information from food packaging, were not tenable.

Even in tracking systems that attempt accessibility, 4 BLV respondents ran into roadblocks preventing them from getting started, with several mentioning the initial pairing procedure requiring a sighted user to read a code on the device. Some physical design choices were hurdles to continued use, such as difficult battery replacement doors or blood test strips relying solely on visual indicators for application.

In some cases, the landscape of available trackers and apps was frustratingly uncharted for the respondents. For example, 1 respondent journeyed through the many pitfalls of attempting to buy an accessible tracker:

Biggest issue is I did not know if an app was going to be accessible until I download it. In the description it does not say whether or not VoiceOver [Apple’s built-in screen reader] is friendly with the app...But sometimes you don’t know if it will work until it is paired to the device which means you have to purchase the device. Then when you try and go and purchase a device especially if you’re doing it online, you don’t know to get the device whether or not it is accessible.

i6fh, CT and TB

Finally, although accessible trackers may be known and available, 9 noted their usual increased costs and inability to afford them. One respondent divulged the relationship between their disability and income and how it affected their device ownership:

Talking devices are a lot more expensive than non-talking ones. Being low income as a totally blind person, I really have to try hard to get those devices.

45jg, CT and TB

Among the respondents who could begin tracking, 49 reported the lack of support for their adaptation tools in the paired apps, with the majority (31/49, 63%) specifically referring to compatibility with screen readers. Some shared incidents of misapplied aid, such as a screen reader identifying a button but using an incorrect label or reading fields in an illogical order. A total of 8 LV respondents mentioned problems with the visibility in apps, wishing for magnification and contrast options, and another LV respondent wanted better braille support.

Two respondents shared different problems with the exercise apps. One LV respondent, who used a magnifier, liked the written descriptions and visual demos via their television but found difficulty during the actual exercise routines when missing verbal cues about new text instructions. Another TB respondent found themselves blocked because of the difficulties in understanding the verbal explanations of the exercises.

Respondents shared the sense of missing out on features that sighted users could use. A total of 19 respondents noted how they were only able to use a subsegment of their tracking systems, such as tracking devices that use inaccessible screens or advanced statistics not available from the accessible portions of the app. In addition, 15 respondents mentioned the frequent use of inaccessible graphs and their ineffectiveness for BLV users.

The immediacy of PHD is sometimes a luxury that is unavailable to BLV users. Some respondents use the aid of others to work around the inaccessible areas of their tracking systems, but when helpers are not available, feedback is delayed. Others experience similar information delays owing to the need to wait until the data are transferred to the accessible portion of the system. One respondent shared annoyance in waiting to read their data:

I had to sync my Fitbit with the Fitbit app in order to access my stats. This was very frustrating as sighted people can just look at their Fitbits to tell how many steps they have done.

7cqb, CT and TB

This knowledge of abled users’ easier experiences in tracking led to varied negative emotions among the respondents. One respondent noted their frustration in the PHD manual entry, in part by measuring themselves against how long a sighted person would take. Another mentioned being jealous of their husband’s enhanced gym experience, as her PHD was locked behind inaccessible screen-based equipment:

I do not recognize it when I am gradually improving by going faster or working harder like my husband does. He says things like “I did my 2 mile run faster today” I have no idea how far I ran...I have no idea if I’m doing better or worse today than yesterday or last week or last month so my motivation is waning.

aeym, CT and TB

At times, the evidence of a better experience came from the respondent’s own history: 1 respondent shared the need to change how they accessed their system because of worsening vision. However, 8 respondents pointed out a deterioration in the accessibility of features in their tracking system, losing access to functionality they previously used.

#### Perceived Low Valuation in Tracking

Some respondents were disinterested in PhD tracking because the benefits did not provide enough value compared to the effort required. Although some reasons may be shared with sighted individuals, in many instances, this valuation was because of their systems’ incompatibility with their situation.

For 10 respondents, this value equation was affected by the extended burden of tracking for BLV users. At times, the data provided by tracking added an additional burden of being reliant solely on memory when reviewing data, perhaps only being presented via the screen reader. Others noted the added time consumption in entering data into apps or simply using the apps in general. The need to involve others in tracking data that required sight was another unwanted burden, as it reduced some respondents’ feelings of independence.

Some respondents shared how the collected PHD from their tracking devices were not trustworthy. Awareness of discrepancies between parallel trackers and devices, incomplete data about known activities, and incompatibility of trackers with ailments such as cerebral palsy were all mentioned as barriers to tracking PHD.

Even if the collected PHD were accurate, the data itself could elicit unfavorable reactions in users. Three respondents found the incoming data too overwhelming, such as 1 CT and TB user who found most notifications as unimportant and resigned to ignore them all because of the difficulty of reading notifications. Moreover, 2 respondents shared concerns with privacy, including a PT and LV respondent who disliked when others were able to read their “personal info” whenever they needed assistance with their Fitbit. For a few respondents, the lucidity of their recorded behavior proved stressful or upsetting; 1 PT and TB respondent preferred “*not wanting to know any bad news*.”

In some PT cases, the tracking experience was not of interest. Three PT respondents merely saw their tracking efforts as an assigned task from others, which once ended meant that they could discontinue further use. In addition, 5 respondents found tracking to be a subtask of their health activity, and when their commitment to the activity dropped, the lack of tracking followed. Moreover, 5 PT respondents found no interest in what they captured (eg, data levels that remained constant throughout), and so their efforts of data entry did not provide enough value for continuance.

### Work-Arounds to Access Inaccessible Data

Some tracking systems aid BLV trackers in adapting to their disability. A majority of respondents (67/132, 50.8%) relied on a screen reader (either on a smartphone or computer) to access the tracked data (Table 2). They augmented inaccessible devices with their smartphones, which provided an accessible screen reader for transferred data, and others relied on the added haptic feedback to alert them to various reminders and alerts. When buttons were mislabeled and screen readers were not helpful, BLV respondents learned by trial and error to memorize the functionalities of the mislabeled buttons.

**Table 2:** How current tracker and past tracker respondents (n=132) access the tracked data based on an optional response question.

	Current tracker (n=69), n (%)	Past tracker (n=63), n (%)	Combined (n=132), n (%)
Screen reader	38 (29)	29 (22)	67 (50.8)
Braille display	0 (0)	1 (1)	1 (0.8)
Magnification or residual vision	0 (0)	8 (6)	8 (6.1)
Assistance from others	3 (2)	4 (3)	7 (5.3)
No response	28 (21)	21 (16)	49 (37.1)

Tracking systems also provided other types of organizational help, such as one user who appreciated how they were able to “*funnel as much as possible into my Apple Health app as the primary hub*,” and another who liked the ability to favorite specific health statistics to add to their feed.

## Discussion

### Summary of the Key Findings and Implications

Self-tracking has been extensively studied in the health informatics community [24-29], but the experience of BLV people was rarely represented. We contributed to an understanding of BLV people’s personal health tracking motivation, practices, needs, and challenges. Our survey showed that BLV respondents have strong desires and needs to track their PHD, and many of them are already tracking their data despite many hurdles. Popular tracking items—exercise, weight, sleep, and food—and the reasons for tracking reported in this study were similar to those of sighted people [3]. However, BLV people experienced diverse accessibility challenges in tracking and accessing data, failing to effectively benefit from self-tracking technologies.

Challenges persist throughout the different phases of self-tracking, starting from identifying accessible tools to difficulty in tracking and reviewing data. The main barriers our respondents experienced included suboptimal tracking experiences and insufficient benefits against the extended burden for BLV people. Some of these accessibility challenges were unique to BLV users, making already burdensome self-tracking even more challenging. For example, a common challenge faced by sighted users was to identify a personal tracking app that would help them meet their goals. To address this problem, sighted users would try out multiple apps before they settle on a tool of their choice—a process called “trialing” [30]. Such an approach is unlikely to be viable for BLV people because it requires considerable efforts to check whether a tool is accessible before they can assess the tool’s efficacy.

### Accessibility Exists on a Spectrum

A recurring theme we identified in the data was whether a technology is accessible is not all black and white, but it exists on a spectrum (refer to the themes under “Knowing It Could Be Better” in Textbox 3). Even if BLV users spot an accessible technology, it is unclear to what extent the technology is accessible: tracking might be possible, but there may be an information delay in accessing the tracked data; data may be accessible only from an app but not from a wearable device, or the feedback (eg, a chart) may not be accessible at all. Contrary to our expectations and to the reported barriers from the open-ended survey responses, most current BLV trackers (59/87, 68%) were satisfied with the personal health technologies that they are using. Of the 34 highly satisfied respondents, 10 (30%) answered the questions specifically for their current setup, that is, they did not consider systems with barriers that had already been pruned from their setup. However, for others, perhaps the contrasting result indicates that despite many accessibility challenges BLV users experience, some people could have successfully used work-arounds to meet their tracking goals or might have been satisfied with subpar experience because they had low expectations to begin with. Further research is warranted to examine the gap between the high level of satisfaction and the accessibility challenges reported and to what extent the currently supported self-tracking features are being used and what key accessibility challenges have high impact on user satisfaction had they been addressed.

Similarly, the relationship between barriers to tracking and people’s current tracking status may prove to be another avenue of research worth investigating further. Although a large majority of NE respondents (22/24, 92%) expressed interest in collecting PHD, additional studies are needed to find more details on the barriers that limit these individuals from beginning to track and possible solutions to overcome them. Although our survey found similar barriers reported by PT and CT members, future work is needed to fully investigate why some persevere when confronted with barriers and others discontinue. It is conceivable that a decreased level of importance assigned to one’s health or circumstantial disadvantages that intensify some barriers are the reasons that contribute to discontinuation, but this inquiry would benefit from studies that allow for more depth beyond this initial survey.

### Limitations and Future Work

Our participants appeared to be technologically savvy, as there were 94.2% (147/156) of smartphone users compared with the US average of 85%, and 96.8% (151/156) laptop or desktop device owners compared with the US average of 77% [31]. Moreover, 44.2% (69/156) of our survey respondents reported that they currently own wearable devices, which is much higher than the US average of 21% [2]. Although not generalizable to all BLV people, this shows our respondents’ high interest in personal health tracking. The challenges we reported regarding PHD tracking still hold because the lived experience of interacting with personal health technologies can come from those who had or have been using the technologies. Furthermore, fewer tech-savvy groups will likely experience the identified accessibility challenges if they were to engage in self-tracking.

However, our findings also indicate that a large variability exists among BLV individuals. Further studies are warranted to understand how general BLV individuals engage in personal health tracking while not conflating the 2 groups—TB and LV people—as their vision capabilities (eg, whether one has residual vision vs not) and respective solutions (eg, GUI based and screen reader based) could be different [32]. Furthermore, we need to devise ways to help BLV individuals with less technical proficiency onboard with self-tracking, such as identifying appropriate tools that meet their individual tracking goals.

We collected data through a web-based and phone survey to recruit more respondents more easily. To ensure the data quality, we carefully inspected the quality of responses and excluded 28 suspicious responses—2 with duplicate IP addresses and 26 with a suspicious pattern. However, as we did not observe or interact with the respondents, the inherent concern about the data quality of web-based surveys remains.

### Conclusions

On the basis of our survey of 156 BLV users’ PHD tracking experiences, we reported quantitative and qualitative findings that contribute to an in-depth understanding of their motivations, tracking practices, challenges, and work-arounds. These findings shed light on design opportunities and areas to focus on making PHD technologies more accessible to BLV individuals. We strongly encourage technology companies and health researchers to invest their efforts in making PHD technologies accessible to BLV people, as a step toward ensuring equitable access to health data for all.

## Appendix

Multimedia Appendix 1 The web-based version of the survey we distributed.

Multimedia Appendix 2 Codebook from the qualitative data analysis.

## Abbreviations

BLV: blind and low-vision
CT: current tracker
GUI: graphical user interface
LV: low vision
mHealth: mobile health
NE: no experience
PHD: personal health data
PT: past tracker
TB: totally blind

## Data Availability

The data sets generated during this study are not available according to our institutional review board protocol and consent form, but the study protocol (questionnaire) and analyses of the qualitative data are available in Multimedia Appendices 1 and 2.
