# Supplementary material for: Personal Health Data Tracking by Blind and Low-Vision People: Survey Study
Source: J Med Internet Res. 2023 May 4;25:e43917. doi: 10.2196/43917 (PMC10196896; doi:10.2196/43917)
Supplement: Multimedia Appendix 1 [file jmir_v25i1e43917_app1.docx]

APPENDIX: SURVEY

Start of Block: Description and Consent Form

Project Title: Understanding how blind and low vision people collect, access, and use the personal health data. 

Purpose of the Study: This research is being conducted by Kyungyeon Lee at the University of Maryland, College Park and colleagues. We are inviting you to participate in this research about personal health data tracking for blind and low vision (BLV) people. The purpose of this research project is to understand how BLV people collect, access, and use their personal health data (e.g., exercise, sleep, diet, heart rate, weight, and blood pressure data) and what obstacles they face in doing so.

Procedures: On the landing page of the online survey, we will briefly explain what the survey is about along with any compensation that you will receive. For the compensation, we will provide Amazon gift cards by raffling. If you want to join the raffle, you can enter your contact information (i.e., email or phone number) at the end of the survey. Participants who win the raffle will be notified by the contact they provided within 30 days from the completion of the data collection. We will give out a $50 Amazon gift card to two participants and a $20 Amazon gift card to ten participants.
Through the survey, we will ask questions about basic demographics (i.e., age, gender, ethnicity, race, education, level of visual impairment). How you collect health data, and the obstacles you go through in obtaining them. You will be asked what kinds of personal health data you track, how and why you track them, and how you access and use the tracked data. We will also collect data on BLV people’s experiences with existing personal health applications or systems. 
The survey may take about 20 minutes to complete. For developing and administering the online surveys, an online survey tool, (UMD) Qualtrics, will be used.

Potential Risks and Discomforts: There is no more than minimal risk. If at any point you are uncomfortable with the survey, you can stop your participation. You may skip ten optional questions you do not wish to answer but cannot skip other mandatory questions. There might be a potential risk of breach of confidentiality—for example, revealing one’s status with visual impairment in case the researcher’s UMD account is hacked. This risk will be mitigated through the procedures described in Section Confidentiality.

Potential Benefits: The research does not have direct benefits to you. The findings, however, could inform the design of future health technologies to be more inclusive of and beneficial to BLV people.


Confidentiality: Only researchers will have access to the raw data stored in password-protected Qualtrics server (university-approved cloud-based survey tool), “UMD Box” (data types: participants’ information -- phone number, email address and audio recordings, research documents, and survey data), and the data will be de-identified for analysis. We may write a report or article about this research project. In doing these, your identity will be de-identified and protected to the maximum extent possible. The collected data will be held for a period of no longer than seven years after the study is completed in the principal investigator’s computer and on the above listed online systems, after which the data will be deleted.

Compensation: We will provide Amazon gift cards by raffling. We will give out a $50 Amazon gift card to two participants and a $20 Amazon gift card to ten participants.

Right to Withdraw and Questions: Your participation in this research is completely voluntary. You may choose not to take part at all. You may also stop participating at any time during the survey, in which case we will remove your data. If you have questions or concerns, please contact the investigator: Kyungyeon Lee, University of Maryland, College Park, kylee@umd.edu, 301-683-8929

Participant Rights: If you have questions about your rights as a research participant or wish to report a research-related injury, please contact: 

University of Maryland College Park Institutional Review Board Office 1204 Marie Mount Hall College Park, Maryland, 20742 
E-mail: irb@umd.edu 
Telephone: 301-405-0678 

For more information regarding participant rights, please visit: https://research.umd.edu/research-resources/research-compliance/institutional-review-board-irb/research-participants This research has been reviewed according to the University of Maryland, College Park IRB procedures for research involving human subjects.

This research has been reviewed according to the University of Maryland, College Park IRB procedures for research involving human subjects. For more information regarding participant rights, please visit: https://research.umd.edu/research-resources/research-compliance/institutional-review-board-irb/research-participants

Statement of Consent: Selecting "I Agree" below indicates that you are at least 18 years of age; you have read this consent form or have had it read to you; your questions have been answered to your satisfaction and you voluntarily agree to participate in this research study. You may save/print a copy of this signed consent form. If you agree to participate, please select "I Agree" below

Q1 Please select your answer below.

- I Agree/Consent (1)
- I Do Not Agree (2)

Skip To: End of Survey If Please select your answer below. = I Do Not Agree

End of Block: Description and Consent Form

Start of Block: Basic Information

Q2 What is your level of visual impairment?

- Totally blind (2)
- Low vision, please simply specify your condition (e.g., light perception, loss of central vision): (1) ________________________________________________
- I don't want to disclosure (5)

Skip To: End of Survey If What is your level of visual impairment? = I don't want to disclosure

Q3 How long have you been in such level of visual impairment?

- Less than 1 year (14)
- 1 year to less than 5 years (15)
- 5 years to less than 10 years (16)
- 10 years or more (but not since birth) (17)
- Since birth (1)
- I don’t know (19)
- Prefer not to say (20)

Q4 Which of the following devices do you personally own? (Check all that apply)

- Desktop computer (1)
- Laptop computer (2)
- Tablet (e.g., Galaxy tab, Apple iPad) (3)
- Smartphone (4)
- Smartwatch or fitness band (e.g., Fitbit, Apple Watch, Galaxy watch) (5)
- Medical device (e.g., Glucometer, heart rate monitor, home blood pressure monitor, thermometer) (6)
- Body fat or body weight scales (8)
- Other (please specify): (7) ________________________________________________

End of Block: Basic Information

Start of Block: Personal Health

Q5 In general, how would you describe your health condition?

- Very Poor (1)
- Poor (2)
- Fair (3)
- Good (4)
- Very Good (5)

Q6 In general, how interested are you in your health?

- Definitely not interested (1)
- Probably not interested (2)
- Neutral (3)
- Probably interested (4)
- Definitely interested (5)

Definition "From now on, you will be asked questions about personal health data." 


In this survey, we use the term “Personal Health Data” to refer to health-related data that people track about themselves outside the clinic. These data include exercise (e.g., step count, miles run), sleep, diet, heart rate, weight, and blood pressure data. We are interested in your experiences of collecting, accessing, and using these data. 


Please answer the following questions.

Q7 Have you ever kept track of your personal health data independent of health professionals?

- Yes, I am currently tracking my personal health data. (1)
- Yes, but only in the past. Currently, I do not track my personal health data. (2)
- No, never. (3)

Display This Question:

If Have you ever kept track of your personal health data independent of health professionals? = Yes, I am currently tracking my personal health data.

Or Have you ever kept track of your personal health data independent of health professionals? = Yes, but only in the past. Currently, I do not track my personal health data.

Q8 What kinds of personal health data do you currently (or did you formerly) track? (Check all that apply)

- Exercise (e.g., step count, miles walked/run) (1)
- Sleep (2)
- Weight (3)
- Food intake (4)
- Water intake (10)
- Sedentary (sitting) time (11)
- Mood (12)
- Stress (8)
- Heart rate (13)
- Blood pressure (14)
- Cholesterol (15)
- Body temperature (20)
- Blood glucose (16)
- Menstrual cycle (19)
- Other (Please specify): (17) ________________________________________________
- None (I currently do not track any personal health data.) (18)

Display This Question:

If Have you ever kept track of your personal health data independent of health professionals? = No, never.

Q9 What kinds of personal health data do you want to track, if any? (Check all that apply)

- None (I don’t want to track anything) (18)
- Exercise (e.g., step count, miles walked/run) (1)
- Sleep (2)
- Weight (3)
- Food intake (4)
- Water intake (10)
- Sedentary (sitting) time (11)
- Mood (12)
- Stress (8)
- Heart rate (13)
- Blood pressure (14)
- Cholesterol (15)
- Body temperature (20)
- Blood glucose (16)
- Menstrual cycle (19)
- Other (Please specify): (17) ________________________________________________

Display This Question:

If Have you ever kept track of your personal health data independent of health professionals? = Yes, I am currently tracking my personal health data.

Or Have you ever kept track of your personal health data independent of health professionals? = Yes, but only in the past. Currently, I do not track my personal health data.

Q10 In addition to what you have been tracking, what other personal health data do you want to track, if any? (Check all that apply)

- None (I don’t have anything else I want to track) (18)
- Exercise (e.g., step count, miles walked/run) (1)
- Sleep (2)
- Weight (3)
- Food intake (4)
- Water intake (10)
- Sedentary (sitting) time (11)
- Mood (12)
- Stress (8)
- Heart rate (13)
- Body temperature (19)
- Blood pressure (14)
- Cholesterol (15)
- Blood glucose (16)
- Menstrual cycle (20)
- Other (Please specify): (17) ________________________________________________

Display This Question:

If Have you ever kept track of your personal health data independent of health professionals? = Yes, I am currently tracking my personal health data.

Q11 How do you track your personal health data, if any? (Check all that apply)

- Paper, like a notebook or journal (1)
- A computer program, like a digital spreadsheet or notepad (2)
- A website or other online tool (3)
- A smartphone app (4)
- A wearable device, like a wristband, clip-on, or smartwatch (5)
- In my head (8)
- Other (Please specify): (9) ________________________________________________

Display This Question:

If Have you ever kept track of your personal health data independent of health professionals? = Yes, but only in the past. Currently, I do not track my personal health data.

Q12 In the past, how did you track your personal health data, if any? (Check all that apply)

- Paper, like a notebook or journal (1)
- A computer program, like a digital spreadsheet or notepad (2)
- A website or other online tool (3)
- A smartphone app (4)
- A wearable device, like a wristband, clip-on, or smartwatch (5)
- In my head (8)
- Other (Please specify): (9) ________________________________________________

Display This Question:

If Have you ever kept track of your personal health data independent of health professionals? = No, never.

And What kinds of personal health data do you want to track, if any? (Check all that apply) != None (I don’t want to track anything)

Q13 If you wanted to track your health data, what would your preferred method be?(Check all that apply)

- Paper, like a notebook or journal (1)
- A computer program, like a digital spreadsheet or notepad (2)
- A website or other online tool (3)
- A smartphone app (4)
- A wearable device, like a wristband, clip-on, or smartwatch (5)
- In my head (8)
- Other (Please specify): (9) ________________________________________________

Display This Question:

If In the past, how did you track your personal health data, if any? (Check all that apply) = A smartphone app

And Have you ever kept track of your personal health data independent of health professionals? = Yes, but only in the past. Currently, I do not track my personal health data.

Q14-1-past What smartphone apps have you used to track which of your data? (optional)

________________________________________________________________

Display This Question:

If How do you track your personal health data, if any? (Check all that apply) = A smartphone app

And Have you ever kept track of your personal health data independent of health professionals? = Yes, I am currently tracking my personal health data.

Q14-1-current What smartphone apps have you used to track which of your data? (optional)

________________________________________________________________

Display This Question:

If In the past, how did you track your personal health data, if any? (Check all that apply) = A smartphone app

And Have you ever kept track of your personal health data independent of health professionals? = Yes, but only in the past. Currently, I do not track my personal health data.

Q14-2-past How was your experience? (optional)

________________________________________________________________

Display This Question:

If How do you track your personal health data, if any? (Check all that apply) = A smartphone app

And Have you ever kept track of your personal health data independent of health professionals? = Yes, I am currently tracking my personal health data.

Q14-2-current How was your experience? (optional)

________________________________________________________________

| Page Break |  |
| --- | --- |

Display This Question:

If In the past, how did you track your personal health data, if any? (Check all that apply) = A wearable device, like a wristband, clip-on, or smartwatch

And Have you ever kept track of your personal health data independent of health professionals? = Yes, but only in the past. Currently, I do not track my personal health data.

Q15-1-past What kinds of wearable devices have you used to track which of your data? (optional)

________________________________________________________________

Display This Question:

If How do you track your personal health data, if any? (Check all that apply) = A wearable device, like a wristband, clip-on, or smartwatch

And Have you ever kept track of your personal health data independent of health professionals? = Yes, I am currently tracking my personal health data.

Q15-1-current What kinds of wearable devices have you used to track which of your data? (optional)

________________________________________________________________

Display This Question:

If In the past, how did you track your personal health data, if any? (Check all that apply) = A wearable device, like a wristband, clip-on, or smartwatch

And Have you ever kept track of your personal health data independent of health professionals? = Yes, but only in the past. Currently, I do not track my personal health data.

Q15-2-past How was your experience? (optional)

________________________________________________________________

Display This Question:

If How do you track your personal health data, if any? (Check all that apply) = A wearable device, like a wristband, clip-on, or smartwatch

And Have you ever kept track of your personal health data independent of health professionals? = Yes, I am currently tracking my personal health data.

Q15-2-current How was your experience? (optional)

________________________________________________________________

| Page Break |  |
| --- | --- |

Display This Question:

If Have you ever kept track of your personal health data independent of health professionals? = Yes, but only in the past. Currently, I do not track my personal health data.

And If

In the past, how did you track your personal health data, if any? (Check all that apply) != In my head

Q17-past How did you access the data you tracked? (e.g., screen reader, smartphone’s text to speech, ask for help) Do you have any accessibility challenges when accessing your data?

________________________________________________________________

Display This Question:

If Have you ever kept track of your personal health data independent of health professionals? = Yes, I am currently tracking my personal health data.

And If

How do you track your personal health data, if any? (Check all that apply) != In my head

Q17-current How did you access the data you tracked? (e.g., screen reader, smartphone’s text to speech, ask for help) Do you have any accessibility challenges when accessing your data?

________________________________________________________________

| Page Break |  |
| --- | --- |

End of Block: Personal Health

Start of Block: Purpose, why?

Display This Question:

If Have you ever kept track of your personal health data independent of health professionals? = Yes, I am currently tracking my personal health data.

Q18 Why are you tracking your personal health data?

________________________________________________________________

Display This Question:

If Have you ever kept track of your personal health data independent of health professionals? = Yes, but only in the past. Currently, I do not track my personal health data.

Q19 Why did you stop tracking your personal health data?

________________________________________________________________

Display This Question:

If Have you ever kept track of your personal health data independent of health professionals? = No, never.

And What kinds of personal health data do you want to track, if any? (Check all that apply) = None (I don’t want to track anything)

Q20 Could you explain why you don't want to collect your personal health data?

________________________________________________________________

Display This Question:

If Have you ever kept track of your personal health data independent of health professionals? = No, never.

And What kinds of personal health data do you want to track, if any? (Check all that apply) != None (I don’t want to track anything)

Q21 Why do you want to collect your personal health data?

________________________________________________________________

End of Block: Purpose, why?

Start of Block: Obstacles

Display This Question:

If Have you ever kept track of your personal health data independent of health professionals? = Yes, I am currently tracking my personal health data.

Q22 Please describe any barriers or challenges you faced when tracking your personal health data.

________________________________________________________________

Display This Question:

If Have you ever kept track of your personal health data independent of health professionals? = Yes, but only in the past. Currently, I do not track my personal health data.

Q23 Please describe any barriers or challenges you faced when you had tracked your personal health data.

________________________________________________________________

End of Block: Obstacles

Start of Block: Technology

Display This Question:

If Have you ever kept track of your personal health data independent of health professionals? = Yes, I am currently tracking my personal health data.

And If

How do you track your personal health data, if any? (Check all that apply) = A computer program, like a digital spreadsheet or notepad

Or How do you track your personal health data, if any? (Check all that apply) = A website or other online tool

Or How do you track your personal health data, if any? (Check all that apply) = A smartphone app

Or How do you track your personal health data, if any? (Check all that apply) = A wearable device, like a wristband, clip-on, or smartwatch

Q24 How satisfied or dissatisfied are you with the current applications or systems you are using to track personal health data?

- Very dissatisfied (1)
- Somewhat dissatisfied (2)
- Neither dissatisfied nor satisfied (3)
- Somewhat satisfied (4)
- Very satisfied (5)

Display This Question:

If Have you ever kept track of your personal health data independent of health professionals? = Yes, but only in the past. Currently, I do not track my personal health data.

And If

In the past, how did you track your personal health data, if any? (Check all that apply) = A computer program, like a digital spreadsheet or notepad

Or In the past, how did you track your personal health data, if any? (Check all that apply) = A website or other online tool

Or In the past, how did you track your personal health data, if any? (Check all that apply) = A smartphone app

Or In the past, how did you track your personal health data, if any? (Check all that apply) = A wearable device, like a wristband, clip-on, or smartwatch

Q25 How satisfied or dissatisfied are you with the applications or systems that you used in the past?

- Very dissatisfied (1)
- Somewhat dissatisfied (2)
- Neither dissatisfied nor satisfied (3)
- Somewhat satisfied (4)
- Very satisfied (5)

End of Block: Technology

Start of Block: Demographic

Q27 Your age

- 18-24 years old (52)
- 25-34 years old (53)
- 35-44 years old (54)
- 45-54 years old (55)
- 55-64 years old (56)
- 65 years old and more (57)
- Prefer not to say (58)

Q28 With which gender do you identify?

- Male (1)
- Female (2)
- Non-binary / third gender (3)
- Prefer not to say (4)
- Prefer to self-describe: (5) ________________________________________________

Q29 What is your race?

- American Indian or Alaska Native (1)
- Asian (10)
- Black or African American (11)
- Native Hawaiian or Other Pacific Islander (12)
- White (13)
- Other (please specify): (7) ________________________________________________
- Prefer not to say (8)

Q31 What is your highest level of educational attainment?

- Less than a high school diploma (1)
- High school degree or equivalent (e.g., GED) (4)
- Some college, no degree (5)
- Associate degree (e.g., AA, AS) (6)
- Bachelor’s degree (e.g., BA, BS) (7)
- Master’s degree (e.g., MA, MS, MEd) (8)
- Professional degree (e.g., MD, DDS, DVM) (9)
- Doctorate (e.g., PhD, EdD) (10)

Q32 What is your current employment status? (Check all that apply)

- Employed full time (40 or more hours per week) (1)
- Employed part time (up to 39 hours per week) (4)
- Unemployed and currently looking for work (5)
- Unemployed and not currently looking for work (6)
- Student (7)
- Retired (8)
- Homemaker (9)
- Self-employed (10)
- Unable to work (11)

End of Block: Demographic

Start of Block: Follow-up interview preference

Q72 What kinds of data do you want to track other than health data, if any? (e.g., smartphone usage time, finance, productivity) (optional)

________________________________________________________________

| Page Break |  |
| --- | --- |

Q73 Would you want to participate in raffling for the Amazon gift card?

- Yes (1)
- No (2)

Display This Question:

If Would you want to participate in raffling for the Amazon gift card? = Yes

Q75 Please enter your contact information (email or phone number).

________________________________________________________________

| Page Break |  |
| --- | --- |

Display This Question:

If Would you want to participate in raffling for the Amazon gift card? = Yes

Q33 Would you be interested in being contacted for future research? If yes, we will send further information through the contact you just provided.

- Yes (1)
- No (2)

Display This Question:

If Would you want to participate in raffling for the Amazon gift card? = No

Q74 Would you be interested in being contacted for future research?

- Yes (1)
- No (2)

Display This Question:

If Would you be interested in being contacted for future research? = Yes

Q34 Thank you for your interests! Please enter your contact information (email or phone number) so that we can contact you for a future study.

________________________________________________________________

End of Block: Follow-up interview preference
