# Supplementary material for: Personal Health Data Tracking by Blind and Low-Vision People: Survey Study
Source: J Med Internet Res. 2023 May 4;25:e43917. doi: 10.2196/43917 (PMC10196896; doi:10.2196/43917)
Supplement: Multimedia Appendix 2 [file jmir_v25i1e43917_app2.pdf]

Codebook from the Qualitative Data Analysis

Tracking doesn't provide enough value	
Tracking gives me new burdens	not wanting to burden others
	reliance on memory
	barrier: burdensome tracking
Tracking doesn't make me feel good about myself	barrier: privacy
	barrier: upsetting data
	barrier: feeling inadequate technically
	barrier: overwhelming data
Activity/tracking isn't worth the effort	health as an assignment
	lacking activity committment
	my data isn't interesting
My data doesn't reflect me	barrier: distrust in tracking accuracy
	tracking less important things

I know tracking could be better	
I'm missing out	barrier: inaccessible graphs
	barrier: information delay
	truncated medical care
	barrier: device accessibility
Tracking doesn't support my adaptation tools	barrier: braille support
	barrier: screen reader
	barrier: visibility
	barrier: misapplied / missing aid
	barrier: general accessibility
Usability hinders my tracking	barrier: device usability
	unidirectional output
	barrier: usability
	sporatic assessibility needs
	barrier: understanding activity
I can't even get started with tracking	barrier: uncharted accessibility
	barrier: physical obstacles
	barrier: unavailable automated tracker
	visual roadblock
	barrier:costs
I know what makes tracking better	wanting to consolidate data
	wanting to expand accessible features
Evidence of a better tracking experience	comparing to abled experience
	accessibility deterioration
	missing out on features

I benefit from tracking data	
I have social reasons for tracking	track: for others
	track: social activity
	track: independence
Tracking helps me learn about myself	track: historical record
	track: curiosity
	track: introspection
	track: diagnostics
	track: progress report
Tracking is a preventative tool	track: family history concerns
	track: age concerns
	track: maintenance
I improve via tracking	track: external goal
	track: encouragement
	track: coaching
	track: improve condition
